# Effects of *Kalimeris indica* powder on Qingtuan (a glutinous rice starch gel food) quality and its potential mechanisms during hydrothermal processing

**DOI:** 10.1016/j.fochx.2025.103198

**Published:** 2025-11-07

**Authors:** Hui Sun, Yuan Liu, Li Fan, Yulin Wang, Xiaojie Zhao, Jinrui Chen, Hanqin Su, Jie Pang, Jianghua Ye, Xiong Liu

**Affiliations:** a College of Tea and Food, Wuyi University, Fujian, Wuyishan, 354300, PR China; b College of Food Science, Fujian Agriculture and Forestry University, Fujian Fuzhou, 350002, PR. China; c College of Food Science, Southwest University, Tiansheng Road 2, Chongqing 400715, PR China

**Keywords:** *Kalimeris indica* powder, Starch gel food, Qingtuan, Hydrothermal processing, Color stability, Starch digestibility, Multi-scale structure

## Abstract

This study investigated the multifaceted effect of *Kalimeris indica* powder (KIP, 0 %–4 %) on Qingtuan, a traditional Chinese glutinous rice starch gel food, during hydrothermal processing. KIP enhanced color stability through chlorophyll encapsulation in a fiber–polyphenol–starch network, and 2 % KIP optimized chewiness (30.41 N·mm) and sensory acceptance (7.4 score), which were associated with modified microstructure, increased short-range ordered structures, and reduced crystallinity. Notably, KIP, rich polyphenols, and soluble/insoluble dietary fibers markedly reduced starch digestibility via physical barrier effects, enzyme inhibition, polyphenol–starch complexation, led to higher resistant starch content (24.8 %), and a lower glycemic index (78.6) at 4 % KIP. Although elevated KIP levels enhanced functional benefits, sensory trade-offs such as bitterness were observed, which necessitated careful optimization. This research demonstrated KIP's potential as a multifunctional, clean-label ingredient for enhancing Qingtuan's nutritional profile, providing insights into starch-bioactive interactions for the development of functional foods.

## Highlights

• KIP-derived chlorophyll significantly enhanced and sustained Qingtuan's green coloration.
• An optimal 2 % KIP improved Qingtuan's chewiness and sensory acceptance.
• KIP's polyphenols and fibers modulated starch digestion, increasing RS and lowering eGI.
• KIP reinforced Qingtuan's starch matrix, increasing short-range molecular order and modifying crystallinity.

## 1 Introduction

Qingtuan, a distinctive glutinous rice starch-based gel food, is deeply cherished in China for its attractive green hue, signature chewy consistency, and unique flavor, particularly during the Qingming Festival (Kong et al., 2023; Wei et al., 2023). Its traditional preparation involves the hydrothermal steaming of a dough made from glutinous rice flour and natural green extracts, typically derived from tender mugwort (*Artemisia argyi*). This steaming process is crucial for achieving complete starch gelatinization and the product's characteristic soft and elastic texture (Choi et al., 2016; Susmitha et al., 2022; Tamura et al., 2019); however, the industrial production of Qingtuan frequently faces ongoing quality and nutritional issues. High-temperature and high-moisture conditions during steaming often accelerate chlorophyll degradation, which leads to undesirable color fading from vibrant green to yellowish-brown (Gong et al., 2019; Yu et al., 2019). Furthermore, as a starch-rich food, Qingtuan typically exhibits a high glycemic response. Its structural integrity can deteriorate post-production, resulting in textural hardening, softening, or surface cracking; this outcome can limit its market potential among health-conscious consumers (Gong et al., 2024).

The fortification of traditional starchy foods with bioactive-rich plant powders has emerged as a promising strategy to enhance their nutritional profiles and physicochemical properties (Poliński et al., 2022). Mugwort is a traditional colorant; however, its widespread use is restricted by a limited seasonal harvest, the notable thermal instability of its pigments, and potential safety concerns for specific consumer groups. These limitations highlight the pressing need for alternative, stable, and functional natural ingredients. *Kalimeris indica*, a widely consumed edible vegetable and traditional herb in southern China, offers a compelling alternative (Ayemele et al., 2021; Huang et al., 2020). It has year-round availability and serves as a rich source of chlorophylls, polyphenols, polysaccharides, and dietary fibers (Anibogwu et al., 2021; Chen et al., 2024; Choi et al., 2016). Notably, phytochemicals such as polyphenols and fibers are extensively documented to interact with starch, influencing its gelatinization, retrogradation, and enzymatic hydrolysis. These interactions can lead to enhanced texture, improved water retention, and reduced starch digestibility by forming complexes or physical barriers within the food matrix (Zhang et al., 2019; Zheng et al., 2024). Thus, *K. indica* powder (KIP) holds potential as a multifunctional ingredient to enhance the quality and nutritional value of Qingtuan.

Despite the well-documented antioxidant and anti-inflammatory properties of *K. indica*, a considerable research gap persists concerning its systematic application within a glutinous rice matrix, particularly under the transformative conditions of hydrothermal processing. Previous studies on plant powder-fortified rice products have often focused on isolated factors, such as enhancing antioxidant capacity or texture (Levickienė et al., 2024; Poliński et al., 2022; Udomwasinakun et al., 2023). However, the complex, multiscale interactions between KIP's diverse bioactive components (e.g., chlorophylls, polyphenols, and fibers) and glutinous rice starch during gelatinization and subsequent gel network formation remain largely uncharacterized. The precise mechanisms by which these complex interactions influence the crystalline structure, short-range molecular order, and microstructure of the starch gel, which are key factors affecting the final texture, color stability, and starch digestibility of Qingtuan, remain unclear. We hypothesize that the KIP components serve as functional fillers and interacting agents, creating a reinforced composite structure that physically obstructs starch chain re-association and chemically interacts with starch molecules. This structure improves quality attributes and delays in vitro digestion.

Therefore, this study aimed to systematically investigate the effects of different concentrations of KIP (0 %–4 %) on the qualitative attributes and in vitro starch digestibility of Qingtuan and elucidate the underlying mechanisms influenced during hydrothermal processing. Qingtuan samples enriched with varying levels of KIP were prepared and comprehensively characterized for their color stability, textural properties, and sensory attributes. To elucidate the fundamental multiscale mechanisms, we analyzed the structural changes of the dough before steaming and Qingtuan after steaming via X-ray diffraction (XRD), Fourier-transform infrared (FTIR) spectroscopy, and scanning electron microscopy (SEM). Furthermore, the effect of KIP on the nutritional profile was assessed by determining the resistant starch (RS) content and calculating the estimated glycemic index (eGI). This research provides novel, mechanistic insights into the starch-bioactive interactions within a traditional food system and offers a scientific basis for the rational design and development of functional glutinous rice products with superior quality, improved health benefits, and increased industrial applicability.

## 2 Materials and methods

### 2.1 Materials and chemicals

#### 2.1.1 Materials

Glutinous rice flour (78.3 % total starch, 7.3 % protein, and 1.2 % [dry basis] lipids) and wheat starch (87.0 % [dry basis] total starch) were sourced from Variety Gold Grain Oil Food Co., Ltd., Henan, China. Fresh *K. indica* aerial parts (86.4 % water, 5.2 % carbohydrate, 3 % protein, 0.9 % lipid, and 1.1 % fiber) were obtained from a local farm in Wuyishan of Fujian Province, China. An amylose/amylopectin kit was purchased from Megazyme (Bray, Ireland). All other chemicals and reagents used were of analytical grade and purchased from Maclin Biochemical Technology Co., Ltd. (Shanghai, China).

### 2.2 Preparation of KIP and enriched Qingtuan

**Fig. 1:**
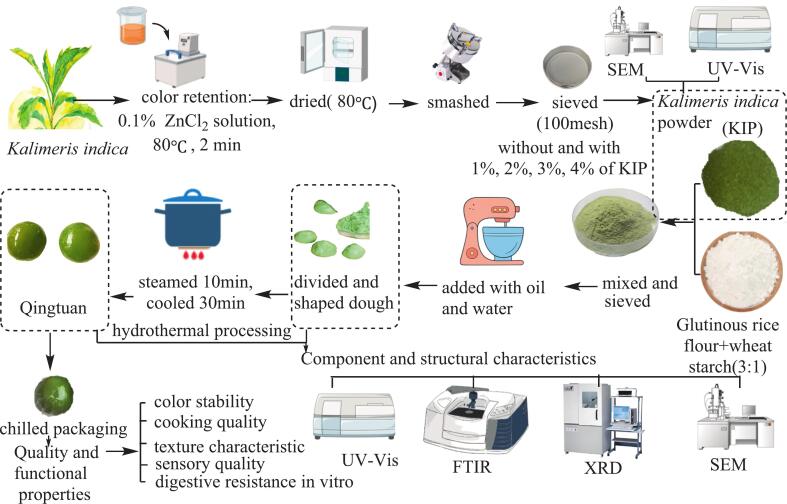
Flowchart of research experimental design and analytical methods.

The flowchart for the preparation of KIP and KIP-enriched Qingtuan is illustrated in Fig. 1. Fresh *K. indica* aerial parts were thoroughly washed with distilled water to remove impurities, followed by blanching in 0.1 % ZnCl_2_ solution at 80 °C for 2 min (material-to-liquid ratio of 1:15) to preserve color and deactivate enzymes. The blanched leaves were immediately cooled in ice water and then dried in a forced-air oven at 80 °C until the moisture content was reduced to below 5 %, achieving the drying endpoint. The dried material was ground into a fine powder using a high-speed universal grinder (WB-100, Beijing Weibochuang Machinery Co., Ltd., China) and sieved through a 120-mesh screen to obtain KIP. The powder was prepared for testing and stored in sealed bags at −20 °C until use.

KIP-enriched Qingtuan samples were prepared by thoroughly mixing glutinous rice flour, wheat starch, sugar flour, and oil (ratio: 30:10:2.4:1.8, *w*/w) with varying proportions of KIP (0 %, 1 %, 2 %, 3 %, and 4 %, based on the total flour weight). Hot distilled water (80 °C) was gradually added to the dry mixture at a ratio of 60 % (*w*/w, based on total solids) while continuously kneading to form a smooth, pliable dough. The dough was portioned, shaped into uniform spheres (approx. 30 g each), and placed on parchment paper in a bamboo steamer. The samples were steamed at 100 °C for 10 min. After steaming, the Qingtuan samples were immediately removed from the steamer and cooled at ambient temperature for 30 min before subsequent analysis or storage at 4 °C (Zheng et al., 2024).

### 2.3 Detection of component contents

The concentrations of chlorophyll *a* (Chl a), chlorophyll *b* (Chl b), and total chlorophyll (TChl) were measured in accordance with a previously reported method (Yu et al., 2019). Chlorophyll retention was determined using the following equations:

(1) $$\text{Chlorophyll retention}\;\left(\%\right)=\frac{\text{total chlorophyll}\;\left(a+b\right)\;\text{of steamed Qingtuan}}{\text{total chlorophyll}\;\left(a+b\right)\;\text{of fresh dough}}\times 100\%$$

The contents of total phenolic compounds (TPCs), free phenolic compounds (FPCs), and bound phenolic compounds (BPCs) were determined following the Folin–Ciocalteu method (Singleton et al., 1965; Ainsworth & Gillespie, 2007) with slight modifications. The contents of total dietary fiber (TDF), soluble dietary fiber (SDF), and insoluble dietary fiber (IDF) were determined in accordance with the AOAC 991.43 method. Total starch content of the samples and amylose content were determined using an amylose/amylopectin kit. The protein and fat contents in different samples were measured following the AOAC, 2000, AOAC 2001.11 and AOAC 2003.05 methods, respectively.

### 2.4 FTIR spectroscopy

The molecular structure and functional groups of Qingtuan samples (raw dough mixture and steamed product) were analyzed through FTIR spectroscopy. Samples were freeze dried and finely ground. The resulting powder was further dried (105 °C for 3 h) before being blended with spectroscopic-grade KBr at a ratio of 1:100 (*w*/w). FTIR spectra were acquired using a Nicolet iS50 FTIR spectrophotometer. Spectra were recorded over the wavenumber range of 4000–400 cm^−1^ with a resolution of 4 cm^−1^ and 16 scans (Zhu et al., 2017).

### 2.5 XRD

The crystalline structure of the freeze-dried Qingtuan powder was determined via powder XRD. Approximately 20 mg sample powder was placed in the sample holder of an X-ray diffractometer (Nicolet 330, Thermo Fisher Scientific, USA). Analysis was conducted using Cu Kα radiation (λ = 0.154 nm) at an operating voltage of 30 kV and a current of 10 mA. Diffraction patterns were scanned over a 2θ range from 5° to 45° at a scanning rate of 8°/min. Origin 2018 software facilitated the computation of relative crystallinity (RC) by using the following formula (Xu et al., 2023):

(2) $$\mathrm{RC}\left(\%\right)=\frac{A_{c}}{A_{c}+A_{a}}\times 100\%$$

where A_c_ and A_a_ represent the areas that correspond to the crystalline and amorphous regions, respectively.

### 2.6 SEM

The microstructure of Qingtuan samples before and after steaming was visualized by SEM. Dried samples were mounted on aluminum stubs using double-sided adhesive tape and sputter coated with a 10 nm-thick gold layer. Micrographs were acquired using a Zeiss EVO18 scanning electron microscope (VEGA3 SBH, Germany) operated at an acceleration voltage of 20 kV. This procedure was adapted from the work of Sun et al. (2025).

### 2.7 Cooking properties

The moisture content of steamed Qingtuan samples was determined in accordance with the standard oven-drying method (AOAC Method 934.01). Precisely weighed samples (3–5 g) were placed in preweighed aluminum dishes and dried in a forced-air oven at 105 °C until a constant weight was attained. Moisture content was calculated as the percentage of weight loss relative to the initial sample weight.

Water absorption capacity (WAC) and cooking loss of steamed Qingtuan samples were determined following the method described by Yang et al. (2021) with slight modifications. Approximately 30 g Qingtuan sample was boiled in 300 mL distilled water for 10 min. Following boiling, the samples were carefully removed, drained, and weighed. The cooking water was collected and dried in a hot air oven at 105 °C to a constant weight to determine the mass of leached solids. WAC and cooking loss were calculated using the following equations:

(3) $$\text{Water absorption}\;\left(\%\right)=\frac{\text{weight of}\;\left(\text{steamed Qingtuan}‐\text{fresh dough}\right)}{\text{weight of fresh dough}}\times 100$$

(4) $$\text{Cooking loss}\;\left(\%\right)=\frac{\text{remaining solid content after drying}}{\text{weight of fresh dough}}\times 100$$

### 2.8 Color measurement and storage stability

The surface color of the Qingtuan samples was measured using a Chroma Meter (Weifu Optoelectronics Technology Co., Ltd., WR-10, Shenzhen, China) with D65 illuminant and a 10° standard observer. L* (lightness), a* (redness/greenness), and b* (yellowness/blueness) values were recorded. Five random spots on the surface of each sample were measured, and the results were averaged (Wang et al., 2022).

For the assessment of color stability during refrigerated storage, each freshly steamed Qingtuan was individually wrapped in plastic wrap and placed in a sealed storage container. The packaged Qingtuan samples were stored in a refrigerator at 4 °C for 3 days. Color measurements (L*, a*, and b*) were conducted at specified time intervals: 0, 3, 6, 12, 24, 48, and 72 h. At each time point, measurements were performed in triplicate on three different Qingtuan samples. The rate of color change was calculated using the following equation:

(5) $$\ln\left[\frac{{\left(-a/b\right)}_{t}}{{\left(-a/b\right)}_{0}}\right]=-k•t$$

where (−a/b)ₜ and (−a/b)₀ represent the chromaticity ratios at time t and 0 h, respectively, and k is the degradation rate constant. All measurements were performed in triplicate on three independent samples per formulation at each time point.

### 2.9 Texture profile analysis (TPA)

The texture properties of the Qingtuan samples were evaluated using a texture analyzer (LS5-Lloyd, Ametek, Inc., USA) equipped with a 12.7 mm cylindrical probe. Each sample was placed on the platform of the texture analyzer for measurement. The test conditions were set as follows: TPA mode, 10 mm/s pretest speed, 10 mm/s posttest speed, 5 gf trigger force, 40 % compression ratio, 15 mm test distance, and 5 s hold time. Hardness, adhesiveness, springiness, and chewiness were determined from the force–time curves. Each Qingtuan sample was tested individually, and each measurement was repeated thrice to ensure accuracy and reliability.

### 2.10 Sensory evaluation

Sensory evaluation of Qingtuan samples was conducted by a panel of 10 postgraduate students (5 males and 5 females; age 20–24 years) specializing in food science and technology. All panelists had prior training in sensory analysis, and they were familiar with the characteristic attributes of traditional Qingtuan. Prior to assessment, all Qingtuan samples were boiled simultaneously for 10 min to ensure uniform preparation. Samples were cut into standardized cubes (2 cm × 2 cm × 2 cm), coded with random three-digit numbers, and served to panelists at ambient temperature on white porcelain plates. The panelists were instructed to cleanse their palates between samples with water crackers (Wei et al., 2023).

A structured 9-point scale (1 = extremely dislike, 9 = extremely like) was used to rate the following attributes: color, overall appearance quality, palatability, smoothness, adhesiveness, chewiness, bitterness/astringency, and overall taste. Attribute definitions and evaluation criteria were developed based on standardized sensory protocols and are summarized in Table S1. In brief, color assessment emphasized uniformity and depth; appearance quality focused on surface smoothness and integrity; palatability was defined as the balance and pleasantness of the chew; smoothness referred to the gloss and tactile qualities of the surface; adhesiveness characterized the stickiness during mastication; chewiness described elasticity and resilience; bitterness/astringency captured undesirable bitter or harsh notes; and overall taste encompassed flavor acceptability. Mean attribute scores and overall acceptability scores were calculated for each sample. Panelists evaluated all samples independently under controlled daylight conditions in sensory booths.

### 2.11 In vitro starch digestion

In vitro starch digestibility was evaluated based on a modified protocol described by Gon˜i, Garcia-Alonso & Saura-Calixto (1997). In brief, steamed Qingtuan samples (0.8 g) were finely ground, combined with 30 mL distilled water, and homogenized by stirring at 150 rpm for 10 min at 37 °C. To simulate the gastric phase, we added 0.8 mL 1 M hydrochloric acid and 1 mL pepsin solution to the mixture, followed by incubation at 150 rpm and 37 °C for 30 min. Gastric digestion was subsequently terminated by the addition of 2 mL 1 M NaHCO₃. Thereafter, 5 mL 0.1 M sodium acetate buffer (pH 6.0) was introduced to adjust and maintain the pH for the subsequent intestinal phase. Immediately, 50 μL amyloglucosidase and 3 mL porcine pancreas α-amylase (both prepared in 0.1 M sodium acetate, pH 6.0) were added, and the final volume was adjusted to 50 mL. Enzymatic hydrolysis proceeded at 37 °C with continuous shaking (200 rpm) for 180 min. At 30 min intervals, 0.8 mL aliquots were withdrawn and mixed with 3.2 mL anhydrous ethanol to terminate enzymatic activity. The mixtures were centrifuged at 10,000 rpm for 10 min (Xiong et al., 2023), and glucose content in the supernatant was determined through the dinitrosalicylic acid method using a UV–Vis spectrophotometer (757 N, INESA, China) at 540 nm. Starch digestibility (%) was calculated as described in Eq. (6). Starch hydrolysis curves were generated for each sample group using Origin 18.0 software.

(6) $$\text{Starch digestibility ratio}=\frac{\text{Content of hydrolyzed glucose}\times 0.9}{\text{Total starch weight}}\times 100\%$$

The kinetics of starch hydrolysis and the digestion rate constant (K, min^−1^) were obtained by fitting the hydrolysis data to a first-order kinetic model (Eq. 4), where t is the duration of digestion, Ct (%) represents the proportion of starch hydrolyzed at a given time, and C∞ corresponds to the maximum extent of starch hydrolysis.

(7) eGI=0.862HI+8.1981

On the basis of these profiles, starch fractions were classified as rapidly digestible starch (RDS, hydrolyzed within 30 min), slowly digestible starch (SDS, hydrolyzed between 30 and 120 min), and RS (fraction remaining unhydrolyzed after 180 min). The hydrolysis index (HI) and eGI were calculated in accordance with the method described by Goni et al. (1997) using Eqs. (8), (9), respectively. Here, HI represents the hydrolysis index, and AUC denotes the area under the hydrolysis curve over the 0–180 min interval.

(8) $$\mathrm{HI}=\frac{\mathrm{AUC}}{{\mathrm{AUC}}_{\text{control}}}$$

(9) eGI=0.862HI+8.1981

### 2.12 Statistical analysis

All experiments were conducted with at least three independent replicates unless otherwise specified. The experimental data are presented as the mean ± standard deviation. Data were subjected to one-way analysis of variance using Origin 18.0 and IBM SPSS version 18.0. The differences among groups were examined using Duncan's multiple-range test. *P <* 0.05 was considered statistically significant.

## 3 Results and discussion

### 3.1 Physicochemical characteristics of KIP and its effect on Qingtuan composition

**Fig. 2:**
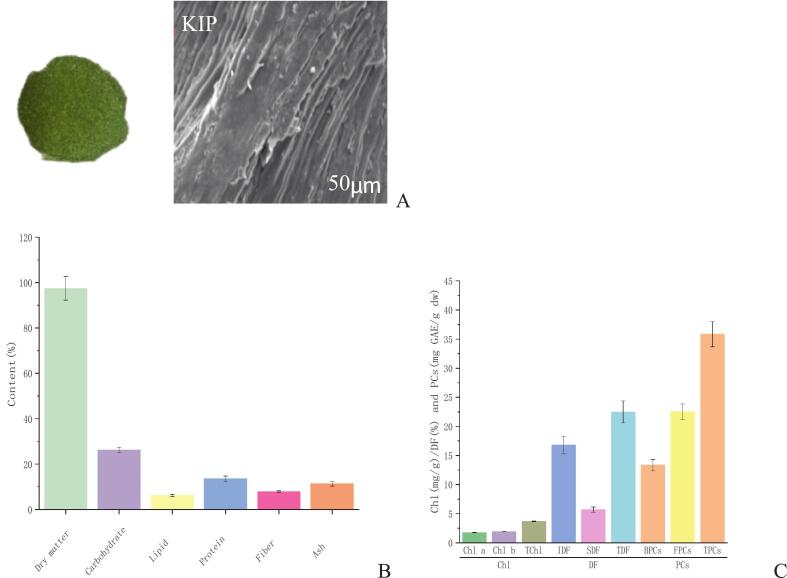
The apparent view, SEM images (A) and chemical composition (B,C) of *Kalimeris indica* powder (KIP). Chl: Chlorophyll, Chlorophyll a: Chl a, Chlorophyll b: Chl b, Total chlorophyll: TChl, phenolic compounds: PCs, Total phenolic content: TPCs, free phenolic compounds: FPCs, and bound phenolic compounds:BPCs, dietary fiber: DF, total dietary fiber:TDF, soluble dietary fiber: SDF, insoluble dietary fiber:IDF. Values are expressed as means ± SD (*n* = 3).

Plant-derived powders are often applied as functional fortificants to improve the nutritional quality of cereal-based products (Park et al., 2021; Wang et al., 2022; Wei et al., 2023). In this study, KIP was incorporated directly into the raw ingredient mixture during the initial dough preparation stage, by blending 1 %–4 % (*w*/w) of KIP uniformly with glutinous rice flour prior to kneading. SEM analysis (Fig. 2A) revealed a complex fibrous microstructure with heterogeneous particle sizes and irregular surfaces, which is characteristic of lignocellulosic matrices rich in dietary fiber. Such a structure may influence KIP's incorporation into and modification of the glutinous rice starch network during hydrothermal processing.

Proximate analysis (Fig. 2B) showed that KIP contained 97.48 % dry matter, including carbohydrates (26.28 %), protein (13.51 %), dietary fiber (11.37 %), lipids (6.19 %), and ash (4.04 %). Bioactive profiling (Fig. 2C) confirmed high pigment levels, with Chl a (22.51 mg/g) dominating over Chl b (3.17 mg/g), TPCs (35.85 mg GAE/g dw), and TDF (22.9 %, ∼70 % insoluble). This composition highlights KIP's potential to enhance the nutritional, functional, and sensory attributes of Qingtuan.

### 3.2 Effect of steaming on pigment, phenolic compounds, and fiber retention

**Fig. 3:**
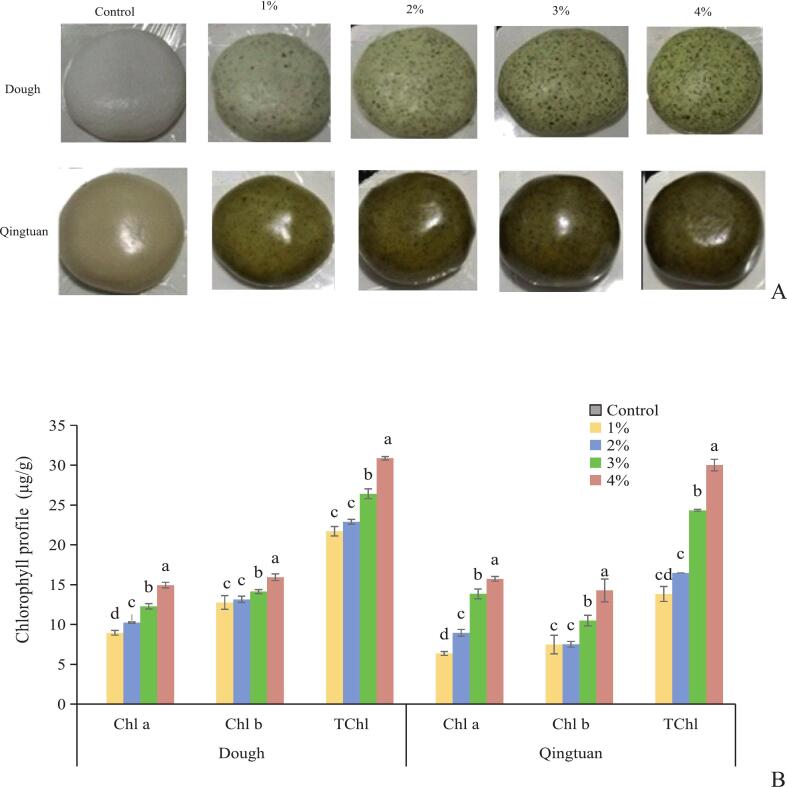

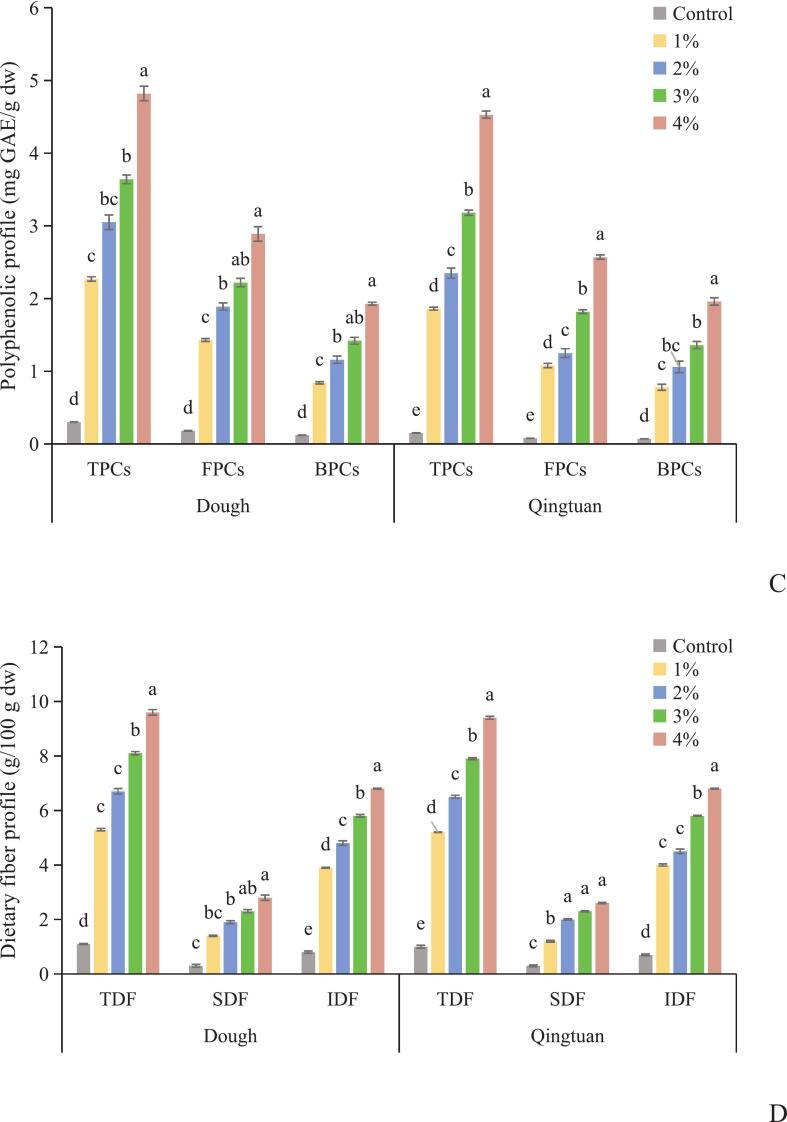
Changes of apparent view (A),chlorophyll (B), polyphenols profile(C) and dietary fiber (D) in Qingtuan before and after steaming. Chlorophyll a: Chl a, Chlorophyll b: Chl b, Total chlorophyll: TChl, Total phenolic content: TPCs, free phenolic compounds: FPCs, and bound phenolic compounds:BPCs, total dietary fiber:TDF, soluble dietary fiber: SDF, insoluble dietary fiber: IDF. Values are expressed as means ± SD (n = 3).Different lowercase letters (a, b, c and d) in the same figure indicate a statistically significant difference (*p* < 0.05).

KIP supplementation induced a dose-dependent shift in product color from pale white (control) to deep green (Fig. 3A), with intensity increasing at elevated levels. Before steaming, Chl a, Chl b, TChl, and phenolic fractions (TPC, FPC, and BPC) increased proportionally with the addition of KIP (Fig. 3B and C), with Chl a being the predominant pigment, reflecting the raw KIP composition.

Steaming significantly (*p* < 0.05) decreased chlorophyll content, with Chl b retention (70 %–75 %) surpassing that of Chl a (55 %–65 %), consistent with their respective thermal stabilities (Yu et al., 2019;Wang et al., 2022). Elevated KIP levels slightly improved pigment retention, which suggests a matrix-mediated protective effect, possibly due to pigment–phenolic–fiber co-localization within the matrix. Phenolic compounds showed overall thermal stability, with no significant changes in TPC, FPC, or BPC for most levels. Retention rates rose with increased KIP addition, which indicates enhanced preservation in fiber-rich systems. Notably, at 4 % KIP, the steamed BPC content slightly exceeded that of unsteamed Qingtuan; this finding suggests that high fortification may promote the heat-assisted release of bound phenolics from cell wall–protein–carbohydrate complexes (Viana et al., 2024), counteracting degradation. This dose-dependent release was absent at low inclusion levels. Dietary fiber was unaffected by steaming (Fig. 3D). KIP supplementation increased TDF from 1.14 % (control) to 9.68 % at 4 % (a 7.49-fold rise), with IDF comprising 70 %–75 % of TDF in all formulations. No significant effect of steaming was observed on TDF, IDF, or SDF, confirming the thermal resilience of structural polysaccharides (Amrani-Allalou et al., 2019; Koubaa et al., 2015).

Therefore, supplemental KIP enhanced Qingtuan with chlorophylls, phenolics, and thermally stable fiber, with phenolic retention improving at elevated levels and bound phenolic content enhancing at 4 %. These effects were likely associated with structural modifications in the starch–protein–fiber matrix during hydrothermal processing.

### 3.3 Molecular interactions assessed by FTIR spectroscopy

**Fig. 4:**
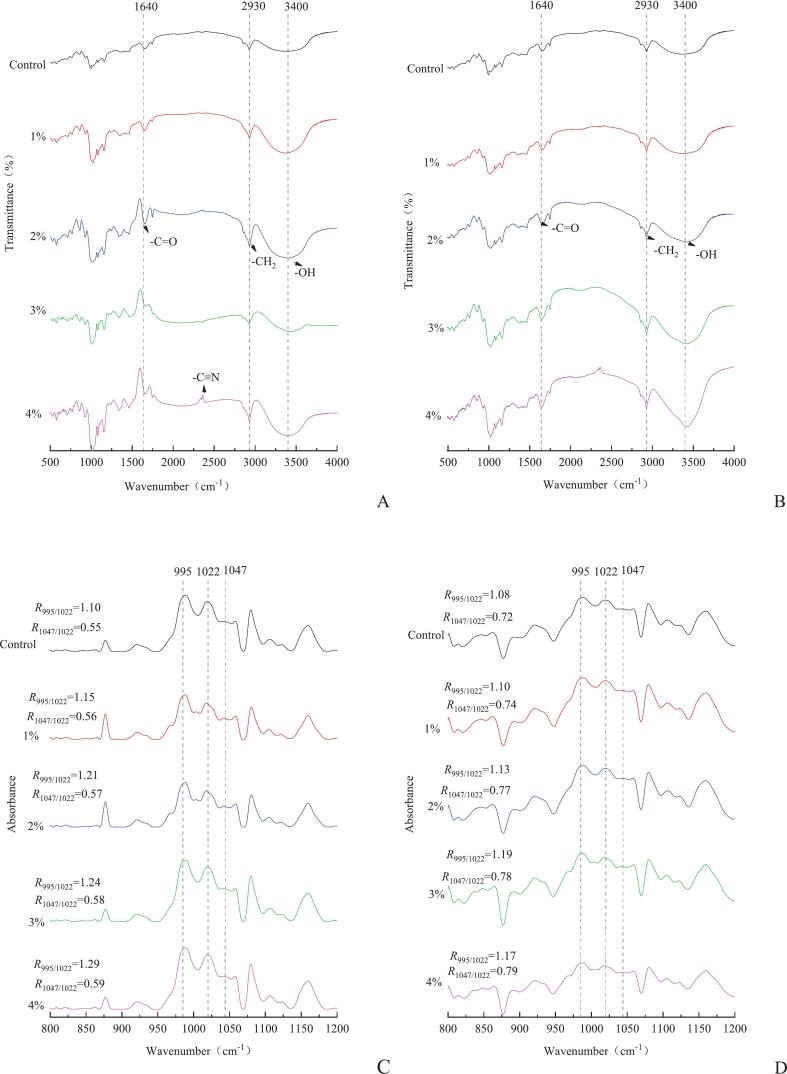
FTIR of the Qingtuan before and after steaming (A:Dough；B:Qingtuan).

The influence of KIP incorporation and steaming on the molecular assembly of Qingtuan was systematically investigated via FTIR spectroscopy, with representative spectra shown in Fig. 4. Throughout the 400–4000 cm^−1^ region, no new absorption peaks nor marked peak shifts emerged upon either KIP addition or thermal processing, confirming that the primary starch–protein–polyphenol matrix was retained without the introduction of novel covalent functionalities (Liu et al., 2018; Shang et al., 2024). Broad absorption at 3000–3650 cm^−1^ corresponded to O—H stretching, providing a sensitive indicator of hydrogen bonding and water interactions in the matrix (Yang et al., 2021).

Prior to steaming, all Qingtuan samples exhibited the typical FTIR signature of starch-based foods: pronounced O—H stretching near 3280 cm^−1^, C—H stretching at 2925 cm^−1^, and dominant bands within the carbohydrate fingerprint region (1200–900 cm^−1^). Incremental increases in KIP content (1 %–4 %) produced discernible enhancements in the absorption at 1047 cm^−1^ (crystalline order) relative to 1022 cm^−1^ (amorphous domains), as well as a slight yet consistent amplification of the 995 cm^−1^ band (double-helix moieties). Calculation of the A₁₀₄₇/A₁₀₂₂ (DO) ratio revealed a steady improvement in short-range molecular order from 1.16 (control) to 1.29 (4 % KIP), whereas A₉₉₅/A₁₀₂₂ (DD) rose from 0.55 to 0.59 (Fig. 4C). This pattern suggests that the introduction of KIP, rich in phenolics and uronic acid polymers, enhanced hydrogen bonding networks and promoted increased local ordering and double-helical association among starch granules, consistent with established reports (Wang et al., 2024; Zhang et al., 2025).

Upon steaming, notable spectral changes were evident for every formulation (Fig. 4B). A substantial decrease in the DO ratio (A₁₀₄₇/A₁₀₂₂) reflected the onset of starch gelatinization and the loss of crystalline lamellar order (Li et al., 2025; Shang et al., 2024). Meanwhile, all samples exhibited an elevation in the DD ratio (A₉₉₅/A₁₀₂₂), attributed to the reformation or partial retrogradation of starch double helices during cooling (Li et al., 2012). Notably, the reduction in short-range order was considerably attenuated in KIP-containing Qingtuan: at 1 %–3 % KIP, DO values remained between 1.10 and 1.19, which are significantly above the control (0.98), with 3 % KIP providing the maximum preservation of order (Fig. 4D). This observation was consistent with the stabilizing effects of KIP-mediated hydrogen bonding and supramolecular interactions, which restricted excessive chain disorder during steaming (Han et al., 2020; Wu et al., 2011). However, further KIP enrichment (4 %) slightly reduced DO, indicating that excessive KIP possbily introduced steric obstacles or segregative effects that limited beneficial ordering (Gong et al., 2019; van Jeroen et al., 1995). Additional spectral features elucidated the mechanisms of matrix reinforcement. The steamed groups, particularly those containing KIP, showed clear widening around 1645 cm^−1^ (amide I), indicating protein unfolding and increased hydration. Similarly, the intensification of a shoulder band at 1735 cm^−1^ suggests the possible formation of ester carbonyls, reflecting either covalent bonding between uronic acid-rich components and matrix hydroxyls or strengthened noncovalent associations (Lee et al., 2023; Zhu et al, 2020).

As a result, FTIR analysis revealed that KIP supplementation significantly improved the structural order and helical architecture of starch–protein matrices before and after thermal processing. This effect was maximized at moderate levels (3 % KIP), supporting the hypothesis that KIP phenolics and polysaccharides participate in supramolecular assembly via hydrogen bonding and, potentially, covalent coupling. These findings were consistent with the evolving paradigm of starch–polyphenol and starch–fiber interactions and directly elucidate the physicochemical mechanisms underlying the observed improvements in Qingtuan quality (Shang et al., 2023).

### 3.4 Crystallinity changes assessed by XRD

**Fig. 5:**
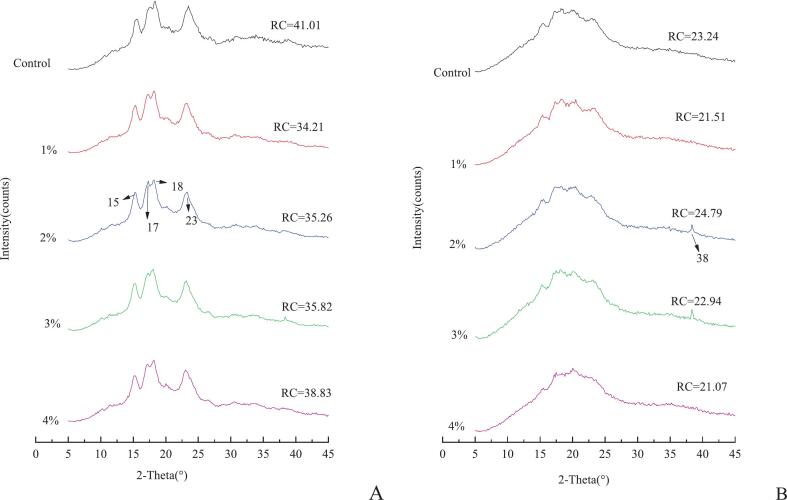
XRD spectrum of the Qingtuan before and after steaming (A:Dough；B:Qingtuan).

XRD patterns revealed significant structural transformations in the starch crystalline architecture of Qingtuan samples due to KIP incorporation and thermal processing (Fig. 5). The diffractograms of unsteamed samples (Fig. 5A) exhibited characteristic A-type crystalline patterns typical of cereal starches, with prominent diffraction peaks at 2θ values of 15°, 17°, 18°, and 23° (Li, 2022; Ren et al., 2021). These peaks corresponded to the double helical crystalline arrangement of amylopectin side chains in native glutinous rice starch. The gradual integration of KIP (1 %–4 %) in unsteamed samples corresponded with a consistent decline in peak intensity, as evidenced by the decrease in RC values from 41.01 % (control) to 38.83 % (4 % KIP). This trend suggests that KIP components, likely phenolics and soluble fibers, initiated partial disruption of the crystalline domains prior to thermal treatment, possibly through intercalation within the starch matrix or through hydrogen bonding with starch hydroxyl groups.

Poststeaming diffractograms (Fig. 5B) demonstrated substantial crystalline structure dissolution across all formulations, characterized by the disappearance of sharp peaks and the emergence of a broad amorphous halo centered around 2θ (20°). This transformation reflects extensive starch gelatinization, where thermal energy disrupts hydrogen bonding in crystalline regions, causing granular disintegration and molecular disordering. The RC values decreased dramatically after steaming, with the control sample exhibiting a reduction from 41.01 % to 23.24 %. Notably, the steamed samples containing KIP displayed concentration-dependent effects on residual crystallinity. All samples showed substantial gelatinization, but the 1 % KIP sample retained slightly less crystallinity (21.51 %) than the control, which suggests an enhanced gelatinization efficiency at low concentration, possibly due to improved heat transfer or water migration facilitated by KIP components. Conversely, at 2 %–3 % incorporation levels, slightly elevated residual crystallinities of 24.79 % and 22.94 % were maintained compared with the 1 % sample. Thus, KIP components may partially inhibit complete starch gelatinization at these intermediate concentrations. A notable observation was the exclusive emergence of a distinct diffraction peak at 2θ = 38° in the 2 % and 3 % KIP samples after steaming. This peak, absent in the control and other KIP concentrations, suggested the formation of a novel crystalline structure at these specific concentration thresholds. This phenomenon likely represented the formation of ordered complexes between KIP components, possibly polyphenols, specific polysaccharides, or mineral microcrystals, and starch molecules that achieved optimal stoichiometry and spatial arrangement at these intermediate concentrations.

The disappearance of this peak at 4 % KIP concentration (21.07 %) indicated that excessive KIP incorporation disrupts the formation of these ordered complexes, possibly due to steric hindrance, competitive binding, or excessive amorphous material introduction that prevents regular crystalline assembly (Wei et al., 2023). This nonlinear concentration effect demonstrated the complex interplay between KIP components and starch molecules during thermal processing (Pan et al., 2025). These XRD results complement the spectroscopic findings and provide structural evidence for the molecular interactions between KIP bioactive compounds and starch during Qingtuan processing. The concentration-dependent effects on crystallinity and the formation of novel ordered structures at specific KIP levels highlight the importance of precise formulation control in optimizing the structural and potentially functional properties of this traditional food product.

### 3.5 Microstructural characterization by SEM

**Fig. 6:**
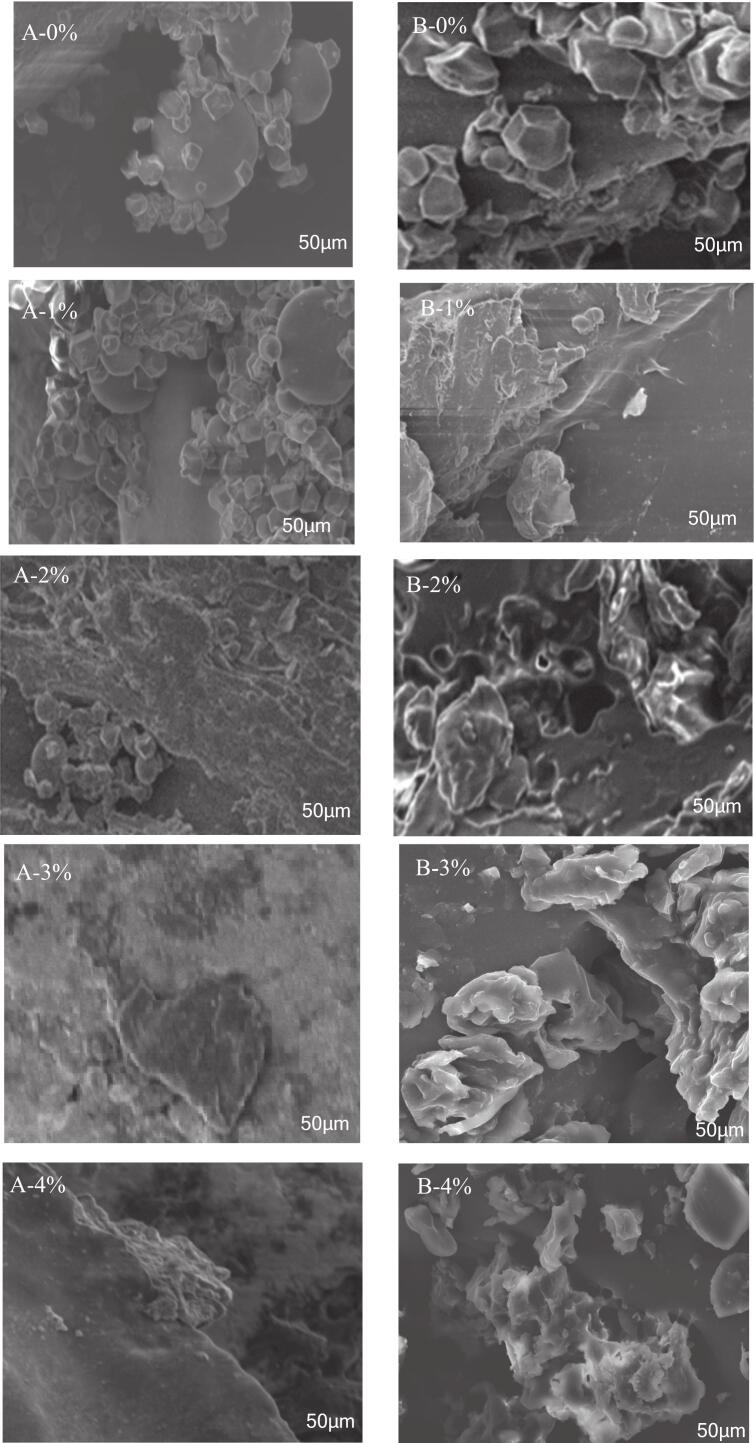
SEM images of Qingtuan with and without KIP(A:Dough；B:Qingtuan).

SEM provided crucial visual insights into the structural modifications induced by KIP incorporation in Qingtuan samples before and after steaming (Fig. 6). Prior to steaming, the control sample (A-0 %) exhibited a densely packed, uniform array of intact and polygonal starch granules, characteristic of native rice flour matrices (Zhang et al., 2025). The introduction of KIP at low concentrations (A-1 % and A-2 %) resulted in fine, amorphous KIP particles dispersed among the granules and subtle irregularities on the starch surfaces, suggesting initial interfacial interactions and potential surface adsorption phenomena (Gangola et al., 2021). Notably, at elevated KIP levels (A-3 % and A-4 %), fibrous aggregates of KIP were prevalent, interweaving with starch to form a heterogeneous and intricate network. This progressive structural complexity suggests that the KIP components acted as fillers and disruptors within the uncooked starch matrix, altering granule packing and potential water channeling pathways (Srikaeo et al., 2018).

Upon steaming, dramatic morphological transitions were observed. The steamed control sample (B-0 %) revealed an extensively swollen, continuous gel matrix with minimal distinction of original granules, demonstrating the classical outcome of starch gelatinization and amylopectin network formation (Obadi et al., 2022). By contrast, steamed Qingtuan with 1 %–3 % KIP (B-1 %, B-2 %, and B-3 %) exhibited a markedly less compact microstructure, with pronounced porosity and residual granular fragments encapsulated within the network. The integration of KIP particles generated microvoids and disrupted the continuity of the gel, suggesting a competitive interaction between starch swelling and fiber network formation. This phenomenon is believed to stem from KIP's dietary fiber impeding complete starch gelatinization and altering water distribution, consistent with the established roles of insoluble fiber in starchy matrices (Gangola et al., 2021; Liu et al., 2025). At 4 % KIP concentration (B-4 %), structural integrity was further compromised by excessive fiber aggregation, resulting in visible discontinuities and phase separation within the matrix. Such structural disorganization may underpin the suboptimal textural and cooking properties observed at elevated KIP levels, as subsequently evidenced by functional measurements (Obadi et al., 2022).

SEM findings collectively underscore that moderate KIP addition (2 %–3 %) fostered the development of a desirable, porous network structure, effectively balancing fiber incorporation and starch gelatinization, whereas excessive KIP led to a structural heterogeneity that is detrimental to gel cohesiveness. These microstructural changes establish a mechanistic basis for the enhanced textural and nutritional properties reported at optimal KIP dosages, and they strongly correlate with FTIR, XRD, and physicochemical analyses.

### 3.6 Consequences for color stability, cooking quality, texture, and sensory properties

#### 3.6.1 Color attributes and stability of KIP-enriched Qingtuan

**Fig. 7:**
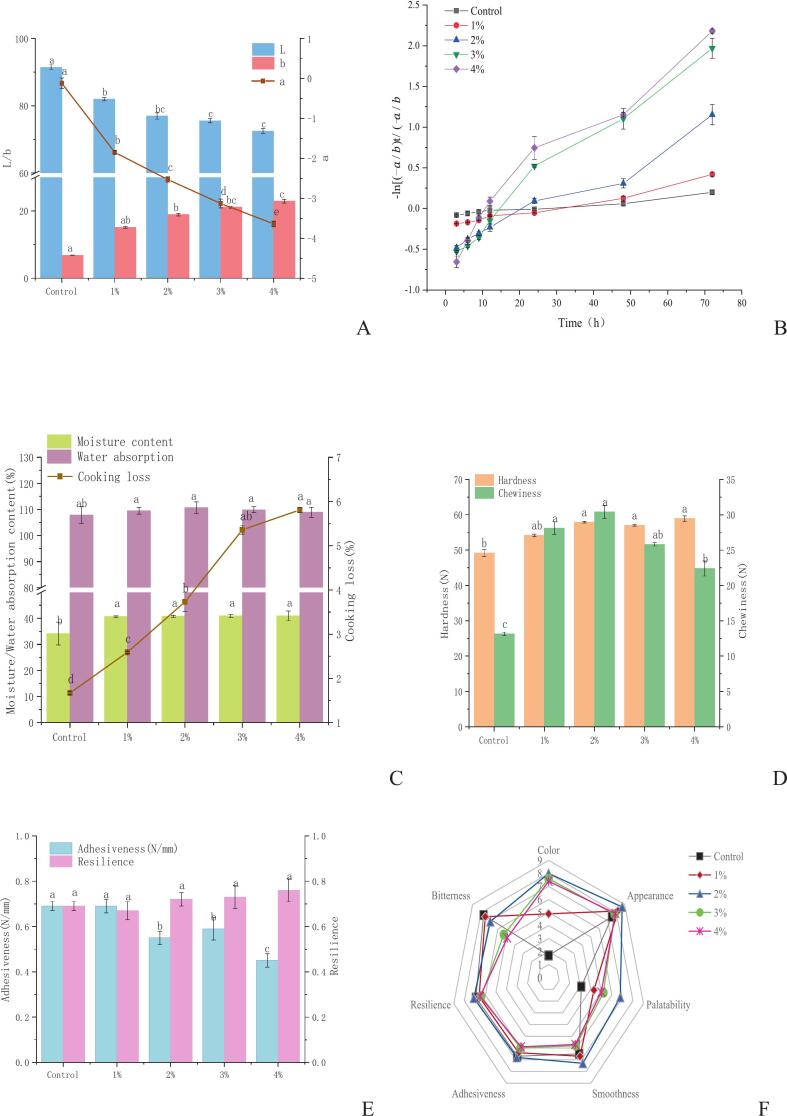
Effect of the addition of KIP on the color parameters (A), rate of discoloration of Qingtuan during storage (B), cooking characteristics (C), textural properties (D,E) and sensory profile (F) of Qingtuan. Data are shown as means ± SD (n = 3). Different letters indicate statistically significant differences (*p* < 0.05).

The incorporation of KIP resulted in a dose-dependent color transformation in Qingtuan, shifting from a pale, grayish-white in the control to a vibrant, deep green (Fig. 3A). This visual change was quantified by significant alterations in CIE Lab* parameters (Figs. 7A). Specifically, the L* value (lightness) progressively decreased from approximately 90 in the control to 72 in the 4 % KIP sample, whereas the -a* (greenness) and b* (yellowness) values markedly increased (*p* < 0.05). This pronounced green coloration was attributed to the inherent chlorophyll content of KIP, as Qingtuan, by itself, typically lacks this vibrant pigment. This complex color profile is a composite effect of pigment addition and microstructural modification (Yu et al., 2019). SEM analysis revealed that KIP was homogeneously embedded within the starch matrix, creating a denser, more compact structure compared with the control. This increased density, combined with the disruption of native starch crystallinity by KIP's amorphous dietary fiber (as suggested by XRD data), likely contributed to the reduced light scattering and the consequent decrease in L* values (Gerza et al., 1999; Zhang et al., 2019).

Beyond the initial appearance, KIP conferred exceptional color stability to Qingtuan during storage at 20 °C for 72 h. The sample supplemented with 1 % KIP exhibited rapid color degradation, retaining only 44.6 % of its initial greenness. By contrast, Qingtuan supplemented with 4 % KIP retained 83.1 % of its green intensity (*p* < 0.05). This protective effect was corroborated by kinetic analysis (Fig. 7B), where the apparent first-order degradation rate constant (k) was significantly reduced from 0.0103 h^−1^ in the control to 0.0025 h^−1^ in the 4 % KIP sample (*p* < 0.05). A similar improvement in color stability through plant-derived additives has been reported in various food systems (Ortiz et al., 2008; Zhu et al, 2020).

The synergistic mechanism underlying this enhanced stability is twofold: physical entrapment and chemical protection. First, the high retention of dietary fiber in KIP facilitated the formation of a robust, cross-linked network with the starch matrix. FTIR spectra confirmed this finding, showing characteristic shifts that indicate extensive hydrogen bonding between the hydroxyl groups of KIP's polyphenols and fibers and the starch chains. This dense network physically confined the chlorophyll molecules, limiting their exposure to pro-oxidative factors such as oxygen and light (Mcevily et al., 1992). Second, the high retention rate of polyphenols ensured a sustained chemical protective environment. These compounds mitigate chlorophyll degradation by scavenging reactive oxygen species and chelating metal ions that catalyze pigment oxidation (Zhu et al., 2020; Yu et al., 2019). Therefore, the enhanced color stability is fundamentally linked to the integrated structural and chemical properties that KIP contributes to the Qingtuan system.

#### 3.6.2 Moisture characteristics and cooking behavior of KIP-enriched Qingtuan

The incorporation of KIP exerted a dual and contrasting effect on the cooking properties of Qingtuan, significantly influencing moisture retention while compromising structural integrity during cooking (Fig. 7C). As illustrated in Fig. 7C, the addition of KIP led to a significant elevation in the moisture content of the steamed Qingtuan, increasing from 34.15 % in the control to 41 % in samples containing 2 %–4 % KIP (*p* < 0.05). This enhancement was mainly due to the high water-holding capacity of the hydrophilic components inherent in KIP, namely, SDFs, and hemicelluloses. These polysaccharides possess abundant exposed hydroxyl groups that readily form hydrogen bonds with water molecules, effectively entrapping them within the food matrix and preventing moisture loss during steaming (Tan et al., 2020). Conversely, the water absorption rate during cooking showed no significant change across all samples (*p* > 0.05). This finding suggests a competitive hydration dynamics: although KIP fibers bind substantial amounts of water, they may simultaneously act as inert fillers, sterically hindering the complete swelling of starch granules and counteracting any potential increase in water uptake during the cooking phase (Pan et al., 2025).

Despite its positive effect on moisture retention, KIP incorporation markedly increased cooking loss in a significant and dose-dependent manner. Cooking loss increased from 1.67 % in the control to 5.81 % at the maximum additive level of 4 % KIP (*p* < 0.05). This harmful effect arises from the disruption of the continuous starch gel network. The particulate nature of KIP interferes with starch–starch and starch–protein interactions, creating discontinuities or “fault lines” within the gel structure (Ferng et al., 2016). During cooking, these weakened zones fail to effectively retain the gelatinized starch granules, leading to the increased leaching of soluble components, primarily amylose and small amylopectin fragments, into the cooking water (Zhang et al., 2019). Therefore, although KIP enhances the overall moisture content, this water may be more loosely bound to the fiber component instead of being fully integrated into a robust, cohesive starch network, which ultimately compromises the structural integrity of Qingtuan upon cooking. The differences in cooking properties are fundamentally linked to the observed alterations in textural hardness (Fig. 7D) and provide a macroscopic basis for the microstructural changes observed in the final product.

#### 3.6.3 Influence of KIP incorporation on the textural attributes of Qingtuan

Textural characterization is a fundamental methodology for objectively quantifying the mechanical and rheological properties of food systems, offering high sensitivity and reproducibility that minimizes the influence of human subjectivity (Chen et al., 2023; Chen et al., 2024). The incorporation of KIP induced significant alterations in the textural profile of Qingtuan, specifically its hardness, resilience, and chewiness (*p* < 0.05; Figs. 7D–7E). A pronounced, dose-dependent increase was observed in the hardness of Qingtuan, which escalated from 49.14 N in the control sample to a peak of 59.51 N in the 4 % KIP-fortified formulation (Fig. 7D). A congruent upward trend was noted for resilience, which also progressively increased and peaked at the 4 % incorporation level. These changes can be primarily attributed to the role of the dietary fiber within KIP, which served as a reinforcing agent within the gelatinized glutinous rice starch network. The fibers likely compete with starch granules for available water, resulting in a concentrated and dense gel structure upon cooking and cooling. Furthermore, the fibers can form hydrogen bonds with polysaccharide chains, physically restricting their mobility and enhancing the overall structural rigidity and elasticity of the Qingtuan matrix (Hou et al., 2023).

By contrast, chewiness exhibited a distinct inverted U-shaped trend, escalating from 13.13 N in the control to a peak of 30.41 N at 2 % KIP, followed by a significant decline at elevated concentrations (Fig. 7D). This complex behavior suggests an optimal reinforcement level for textural integrity. At low concentrations (≤2 %), the KIP fibers were well-integrated, and the increase in chewiness was synergistically driven by the enhanced hardness and resilience. However, beyond this optimal point, excessive, insoluble KIP particles may compromise the continuity and homogeneity of the starch–protein gel network. These particles can act as structural defects or “weak points,” creating a phenomenon of phase discontinuity. This condition leads to a product that, although challenging to compress initially, becomes increasingly brittle and less cohesive, thereby reducing the total energy required for mastication and lowering the chewiness value (Park et al., 2021). Notably, the adhesiveness of the samples remained statistically unchanged (*p* > 0.05) across all treatments (Fig. 7E), indicating that KIP incorporation did not significantly alter the surface properties of the Qingtuan that determine adhesion.

#### 3.6.4 Sensory profile of KIP-enriched Qingtuan

The radar chart (Fig. 7F) clearly visualized the sensory evaluation scores for Qingtuan formulated with varying KIP concentrations (0 %–4 %). The addition of KIP significantly improved the visual and aromatic attributes of the product. The scores on color and appearance increased significantly (*p* < 0.05) with KIP concentration, which was directly attributable to the natural chlorophylls in the powder, imparting the characteristic and desirable vibrant green hue. Similarly, aroma scores were enhanced, peaking at the 2 % concentration, suggesting that the volatile compounds native to KIP contributed a pleasant, fresh herbaceous note that was well-received by the panelists.

A complex, nonlinear relationship was observed for the sensory attributes related to texture and taste. The panelists' perception of decreasing smoothness and resilience at the 4 % KIP level correlated directly with the instrumentally measured increase in hardness (Fig. 7D). At this high concentration, the fibrous particles likely became perceptible in the mouth, contributing to a less desirable, slightly gritty mouthfeel. This finding was consistent with studies where high levels of insoluble fiber negatively affected the textural smoothness of starch-based gels (Tan et al., 2020). Similarly, taste parameters, including palatability and bitterness, indicated an optimal level at 2 % KIP. Beyond this concentration, palatability scores declined significantly, which coincided with a sharp increase in perceived bitterness. This outcome is a classic dose–response effect of phenolic compounds, which are abundant in KIP; at high concentrations, their inherent bitterness becomes dominant and detracts from the overall flavor profile.

Consequently, the overall acceptability score exhibited a parabolic trend, significantly increasing from the control to a peak at 2 % KIP incorporation (7.4) before declining at the 4 % level. This result demonstrates that the 2 % KIP formulation achieved an optimal sensory balance, maximizing the positive contributions to color and aroma and enhancing texture without introducing the negative attributes of grittiness and bitterness. This result, which supported the physicochemical and textural analyses, highlights the critical relationship between precise ingredient formulation and final consumer approval in the development of functional foods (Wang et al., 2022).

### 3.7 In vitro starch digestibility analysis modulation of starch digestibility and glycemic properties

**Fig. 8:**
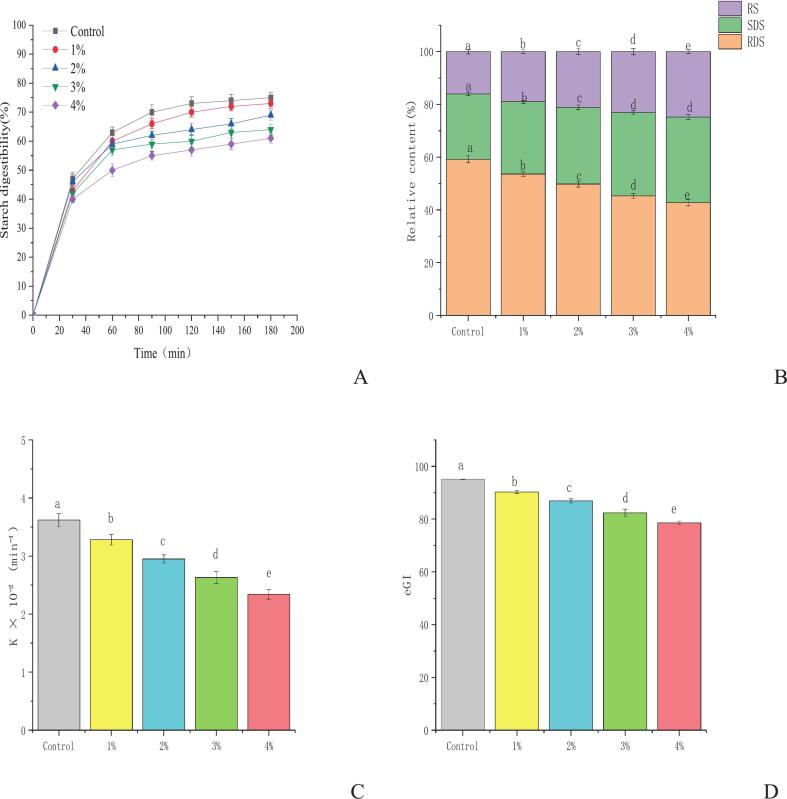
Digestion curves (A) of Qingtuan with different levels of KIP; relative proportions of rapidly digestible starch (RDS), slowly digestible starch (SDS), and resistant starch (RS) (B); kinetic constant (k) of starch hydrolysis (C); and expected glycemic index (eGI) values (D) of Qingtuan. Values are expressed as means ± SD (n = 3). Different letters indicate significant differences among samples (*p* < 0.05). Control: Qingtuan without KIP addition; 1 %, 2 %, 3 %, 4 %: Qingtuan samples containing increasing levels of KIP.

**Fig. 9:**
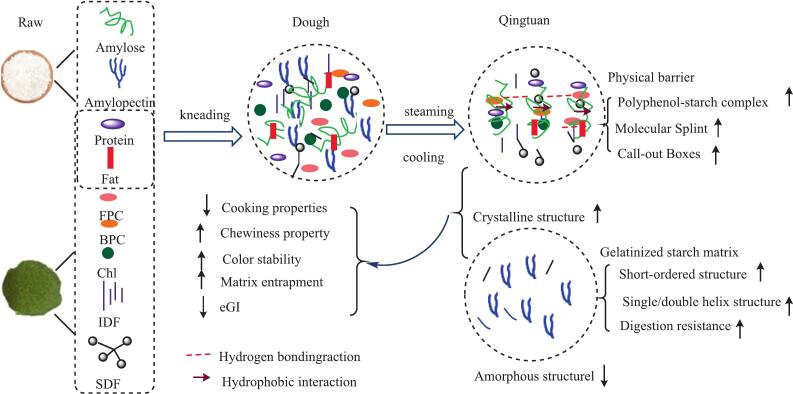
Schematic illustration of the steam-driven interplay between starch, pigment, phenolic and fiber in KIP-enriched Qingtuan dough.

The incorporation of KIP significantly altered the starch digestibility of the Qingtuan product in a dose-dependent manner. The control Qingtuan, prepared without KIP, exhibited the highest rate and extent of starch hydrolysis, reaching a final digestibility of 75 % after 180 min (Fig. 8A). This rapid digestion is characteristic of gelatinized rice starch and corresponds to a high proportion of RDS (59.2 %) and a minimal content of RS (16.0 %), as illustrated in Fig. 9B. Conversely, the addition of KIP consistently inhibited starch hydrolysis. This inhibitory effect was intensified with increasing KIP concentration, with the 4 % KIP formulation showing the lowest final digestibility of 61 %. This observed reduction in starch hydrolysis was significantly influenced by the action of key KIP bioactives: dietary fibers and polyphenols. This reduction in overall digestibility was attributed to a remarkable compositional shift in starch fractions. Specifically, KIP addition induced a progressive and significant (*p* < 0.05) decrease in the RDS fraction, which decreased to 42.7 % in the 4 % KIP sample. Concurrently, the RS fraction, which resists enzymatic breakdown in the small intestine, nearly doubled from 16.0 % in the control to 24.8 % in the 4 % KIP sample (Fig. 8B). The conversion of RDS to RS is a crucial process for improving the nutritional profile of starchy foods. The underlying mechanisms for these observed effects are likely due to the synergistic actions of KIP's major components—particularly its dietary fibers and polyphenols—which contribute to physical barrier effects around starch granules, direct enzyme inhibition, and polyphenol–starch complexation, collectively impeding amylase accessibility and catalytic action (Cai et al., 2023; Paul et al., 2020). This “encapsulation effect” is a recognized strategy for mitigating the glycemic response of starch-based foods.

The kinetic parameters derived from a first-order model further elucidated the mechanism by which KIP attenuates starch digestion. As shown in Fig. 8C, the hydrolysis rate constant (k) decreased significantly and linearly from 3.62 × 10^−2^ min^−1^ in the control to 2.34 × 10^−2^ min^−1^ in the 4 % KIP sample. This reduction in k indicated a reduced enzymatic degradation of starch, supporting the trends observed in the hydrolysis curves. Consequently, the eGI, a crucial predictor of the postprandial glycemic response, was markedly reduced (*p* < 0.05) by KIP fortification (Fig. 8D). The eGI value for the control sample was 95.1, classifying it as a high-GI food, whereas the addition of 4 % KIP lowered the eGI to 78.6, reclassifying the product into the medium-GI category. The significant reduction in glycemic potential is a direct outcome of the decreased rate of digestion (lower k) and the reduced amount of available glucose (lower RDS, higher RS). The formation of a robust and enzyme-resistant food matrix is central to this phenomenon. The bioactive compounds in KIP, particularly flavonoids and other polyphenols, likely interacted with starch polymers (amylose and amylopectin) via hydrogen bonds and hydrophobic interactions during cooking and cooling. These interactions can inhibit starch retrogradation into digestible forms and instead promote the formation of enzyme-resistant structures, such as polyphenol–starch complexes, thereby increasing the RS content (Xiong et al., 2023). Furthermore, polyphenolic compounds, including flavonoids and phenolic acids, are effective inhibitors of α-amylase and α-glucosidase, directly reducing the catalytic efficiency of these key digestive enzymes (D'Costa & Bordenave, 2021). Collectively, these results demonstrate that KIP acted through many physical and biochemical mechanisms to produce a Qingtuan product with enhanced nutritional properties and reduced glycemic impact.

### 3.8 Potential mechanisms for the fortifying effect of KIP on Qingtuan quality

The potential mechanisms by which KIP fortification facilitates the diverse improvements in Qingtuan are theoretically illustrated in Fig. 9. The observed improvements in physicochemical, textural, and nutritional profiles are not merely additive but arise from a series of hierarchical interactions among KIP's functional components, specifically dietary fibers, polyphenols, and chlorophyll, and the native waxy rice starch matrix, progressing sequentially from dough formation to hydrothermal processing and cooling.

The process is initiated during the kneading phase, where the system's architecture is fundamentally re-engineered. KIP's components, particularly its insoluble fibers and diverse phenolic compounds, are thoroughly integrated within the starch–protein network. This active molecular integration of KIP constituents into the starch–protein matrix, rather than a passive physical mixing, was confirmed by FTIR analysis, which demonstrated that the abundant hydroxyl groups on KIP's polyphenols and fibers form extensive hydrogen bonds with starch chains. Such integration-driven pre-organization of the dough matrix results in a cohesive and tightly packed structure that regulates water distribution and mobility even before heat is applied, a phenomenon observed in other fiber- and polyphenol-rich composite dough systems (Gangola et al., 2021).

Upon hydrothermal processing (steaming), this preconfigured system undergoes a controlled transformation. KIP components act as functional modulators, interfering with the complete hydration and swelling of starch granules by competing for available water and physically encasing the granules. This interference, acting as a “molecular splint” (Fig. 9), hinders amylose leaching and maintains a degree of granular integrity. This mechanism is strongly supported by our SEM observations, which showed reduced starch granule disintegration, and by XRD data indicating increased residual crystallinity in fortified samples. The resulting structure is a heterogeneous, reinforced composite gel, rather than the homogenous, fully gelatinized matrix of the control. The change in starch gelatinization behavior by plant-derived fibers and phenolics is a well-documented strategy for constructing starch-based foods (Gutierrez-Osnaya et al., 2020; Zeng et al., 2024).

This engineered matrix architecture is central to the observed functional enhancements. First, the dense, cross-linked network offers a superior physical barrier that encapsulates chlorophyll molecules. This “matrix entrapment” effect limits their exposure to pro-oxidative factors, such as oxygen and light, acting synergistically with the chemical antioxidant activity of *co*-localized polyphenols to dramatically improve color stability (Mcevily et al., 1992). Second, the textural properties are directly influenced by this reinforced network. The consistent improvement in hardness with KIP addition is due to the role of fibers as a reinforcing filler, which enhances structural rigidity (Huang et al., 2020). The inverted U-shaped trend between chewiness and sensory acceptability revealed a dose-dependent optimum at 2 %. Beyond this point, excessive insoluble particles likely disrupted the gel's integrity, resulting in structural defects that increased hardness but decreased cohesive energy, a common challenge in functional food formulation (Chen et al., 2023).

Furthermore, the presence of KIP directs starch retrogradation during cooling toward a less digestible architecture, profoundly affecting the nutritional profile. The formation of noncovalent polyphenol–starch complexes via hydrogen and hydrophobic bonds, a key mechanism shown in Fig. 9, which sterically hinders the re-association of starch chains with digestible structures and instead promotes enzyme-resistant forms (Xiong et al., 2023). Concurrently, KIP's polyphenols are known potent inhibitors of α-amylase and α-glucosidase, directly reducing the catalytic efficiency of carbohydrate digestion (Dang et al., 2023; D'Costa & Bordenave, 2021). Altogether, these mechanisms of promoting the formation of ordered, enzyme-resistant structures (i.e., RS3) and direct enzyme inhibition are responsible for the significant reduction in the eGI. Thus, KIP fortification transformed the Qingtuan matrix from a molecular to a macroscopic level, creating an integrated system where structural reinforcement, chemical protection, and modulated starch digestibility are intricately interconnected to yield a superior and healthy food product.

## 4 Conclusion

In this study, the effects of KIP (0 %–4 % *w*/w) on the physicochemical, structural, sensory, and nutritional properties of Qingtuan were systematically investigated. Our findings reveal that KIP effectively facilitated the retention of its chlorophyll content within robust fiber–polyphenol–starch networks, resulting in sustained green coloration across hydrothermal processing in a dose-responsive manner. A distinct textural optimum was identified at 2 % KIP incorporation, which provided the most favorable balance of chewiness and overall liking without imparting excessive hardness at higher concentrations. Structural analyses through FTIR and XRD demonstrated that KIP addition increased short-range molecular order (R₁₀₄₇/₁₀₂₂ from 1.10 to 1.29) and enhanced double-helix content (from 0.55 to 0.59) prior to steaming, with partial preservation of these ordered domains after thermal processing. These improvements were attributed to hydrogen bonding and hydrophobic interactions between KIP polyphenolics/ fibers and starch helices, as confirmed by SEM observations of a dense microstructure. In vitro digestibility analysis showed a marked shift from RDS toward SDS and RS and a lower predicted glycemic potential, consistent with mechanisms driven primarily by KIP polyphenols and fibers. These beneficial effects, encompassing processing-resilient pigment retention and considerable modulation of starch digestibility, directly address the inherent limitations of traditional waxy rice matrices, such as heat-labile green coloration and rapid starch hydrolysis. Consequently, KIP-enriched Qingtuan aligns well with contemporary objectives for functional food development. The concentration-dependent effects revealed critical insights into product development, allowing manufacturers to balance enhanced functional/nutritional benefits against slight sensory limitations at elevated KIP levels. Future research should focus on the optimization of KIP particle size and processing parameters to enhance bioactive retention while minimizing off-flavors, evaluating in vivo glycemic responses, and exploring the application of KIP in other starch-based food systems to expand its potential in functional food formulations.

## CRediT authorship contribution statement

**Hui Sun:** Writing – original draft, Project administration, Investigation, Funding acquisition. **Yuan Liu:** Formal analysis, Data curation. **Li Fan:** Project administration, Funding acquisition. **Yulin Wang:** Project administration. **Xiaojie Zhao:** Data curation. **Jinrui Chen:** Formal analysis, Data curation. **Hanqin Su:** Data curation. **Jie Pang:** Writing – review & editing, Conceptualization. **Jianghua Ye:** Writing – review & editing, Project administration. **Xiong Liu:** Writing – review & editing.

## Declaration of competing interest

The authors declare that they have no known competing financial interests or personal relationships that could have appeared to influence the work reported in this paper.

## Data Availability

Data will be made available on request.

## Appendix A Supplementary data

Supplementary material Image 1
