# Supplementary material for: Effects of Kalimeris indica powder on Qingtuan (a glutinous rice starch gel food) quality and its potential mechanisms during hydrothermal processing
Source: Food Chem X. 2025 Nov 7;32:103198. doi: 10.1016/j.fochx.2025.103198 (PMC12664087; doi:10.1016/j.fochx.2025.103198)
Supplement: Supplementary file 1 — Supplementary material [file mmc1.docx]

Table S1 Effect of KIP addition on the sensory characteristics standard of Qingtuan

| Evaluation Criteria (Score) | Rating Standard | | |
| --- | --- | --- | --- |
|  | Excellent (7-9) | Good (4-6) | Poor (1-3) |
| Color | Uniform color distribution with deep pigmentation | Slightly uneven color distribution, moderate pigmentation | Irregular coloration with pale appearance |
| Appearance | Intact spherical shape, smooth surface, uniform size | Complete shape with rough surface texture, uniform size | Deformed structure, rough surface, non-uniform size |
| Mouthfeel | Pleasant mouthfeel with high palatability | Moderate palatability | Unpleasant texture with poor acceptability |
| Smoothness | Glossy surface with pronounced smoothness | Moderately smooth surface with faint luster | Matte surface with noticeable roughness |
| Stickipness | Optimal viscosity without dental adhesion | Increased viscosity causing slight adhesion | Excessive viscosity with strong adhesion to teeth |
| Chewiness | Appropriate elasticity with resilient texture | Reduced elasticity requiring minimal mastication effort | Low elasticity with mushy texture |
| Bitter/Astringent Taste | Absence of bitter/astringent aftertaste | Slight detectable bitterness | Pronounced bitter/astringent flavor |
